# Complete Genome Sequence of the Diatom-Associated Bacterium *Sphingorhabdus* sp. Strain SMR4y

**DOI:** 10.1128/MRA.00482-19

**Published:** 2019-07-18

**Authors:** Mats Töpel, Matthew I. M. Pinder, Oskar N. Johansson, Olga Kourtchenko, Anna Godhe, Adrian K. Clarke

**Affiliations:** a Department of Marine Sciences, University of Gothenburg, Gothenburg, Sweden; b Gothenburg Global Biodiversity Centre, Gothenburg, Sweden; c Department of Biological and Environmental Sciences, University of Gothenburg, Gothenburg, Sweden; Georgia Institute of Technology

## Abstract

The bacterial strain SMR4y belongs to the diverse microbiome of the marine diatom Skeletonema marinoi strain R05AC. After assembly of its genome, presented here, and subsequent analyses, we placed it in the genus *Sphingorhabdus*. This strain has a 3,479,724-bp circular chromosome (with 3,340 coding sequences) and no known plasmids.

## ANNOUNCEMENT

In an effort to identify microbiome constituents of the chain-forming diatom Skeletonema marinoi, various associated bacteria are being isolated, sequenced, and characterized (1–5). Here, we report the genome sequence of strain SMR4y, isolated from a culture of *S. marinoi* strain R05AC. Strain SMR4y has been found in two separate isolates of *S. marinoi* obtained at different times and geographical locations, has been in coculture with *S. marinoi* for almost a decade following its field isolation, and has the ability to stimulate growth of *S. marinoi* when added in excess (O. N. Johansson, M. I. M. Pinder, F. Ohlsson, J. Egardt, M. Töpel, and A. K. Clarke, unpublished data). For these reasons, we believe strain SMR4y to be a member of the *S. marinoi* microbiome, as opposed to a random cocultured bacterium.

The diatom culture was established from a revived resting cell taken from top-layer sediment at 14-m depth in May 2010 from Öresund, Sweden (55°59.16′N, 12°44.02′E); collection was performed using a box corer. The diatom culture was maintained in f/2-Si medium and grown at 16°C. Isolation of strain SMR4y was performed at the same temperature in darkness by spotting and iterative dilution streaking on marine agar plates; strain SMR4y was sampled from one of the resultant colonies. The colony was grown overnight at 30°C in 50 ml Marine broth 2216 (Difco BD, USA) prior to DNA extraction; plant DNAzol reagent (Invitrogen Life Technologies, USA) was used to extract genomic DNA as per the manufacturer’s instructions. Sequencing was performed on the PacBio RS II platform (Pacific Biosciences, Menlo Park, CA, USA) using one single-molecule real-time (SMRT) cell, producing 163,482 unfiltered reads (950.9 Mbp total). Filtering was performed using the SMRT Portal version 2.3.0 P_Filter module to remove reads with quality lower than 0.80 and/or reads shorter than 100 bp and subreads shorter than 500 bp (6) (unless otherwise specified, all software was run using default settings). This resulted in 68,619 reads (413.7 Mbp total) that were used in the assembly. Genome assembly was performed using Canu version 1.3 (7) (genome size parameter, 4.5 m). The first 7,058 bp of the assembled contig, found using a BLASTn search (8) to be highly similar to the end of the contig, were trimmed to enable contig circularization. This was confirmed by joining the ends and realigning the reads using the SMRT Portal version 2.3.0 RS_Resequencing.1 protocol (Pacific Biosciences) (6); this protocol includes a correction step using the Quiver algorithm (6). The result was a single chromosome of 3,479,724 bp, with a G+C content of 58.0% and an average read coverage of 100.73× (results summarized in Table 1). This is currently the only *S. marinoi* strain R05AC-associated bacterium not found to contain plasmids, meaning that they are either absent from this bacterium or are smaller than 10,000 bp and therefore were removed during the sequencing library preparation step.

**TABLE 1:** Summary of the *Sphingorhabdus* sp. strain SMR4y assembly and annotation

Characteristic	Value
Assembly	
No. of reads (filtered)	68,619
Bases (filtered) (bp)	413,668,254
Overlapping bases trimmed from start of contig (bp)	7,058
Final circular contig size (bp)	3,479,724
G+C content (%)	58.0
Avg read coverage (×)	100.73
Annotation (no.)	
CDSs	3,340
Pseudogenes	4
tRNAs	46
rRNAs	6
ncRNAs	4
tmRNAs	1

Prokka version 1.12beta (9) was used to annotate the strain SMR4y genome, inferring 3,340 coding sequences (CDSs) (2,742 with a functional prediction), 4 pseudogenes, 46 tRNAs, 6 rRNAs, 4 noncoding RNAs (ncRNAs), and 1 transfer-messenger RNA (tmRNA) (results summarized in Table 1). Two identical 16S rRNA sequences are present on the chromosome; these have 99.9% and 99.4% identity, respectively, to partial sequences from Sphingorhabdus flavimaris strains R-36742 and SW-151^T^ (GenBank accession numbers FR691421 [1,441 bp] and NR_025814 [1,444 bp], respectively). The SMR4y sequence was trimmed to the same length for this comparison, with 99.7% identity to the two identical full-length 16S sequences of *Sphingorhabdus* sp. strain YGSMI21 (GenBank accession number NZ_CP022548) and 99.5% identity to the two identical full-length 16S rRNA sequences of *Sphingorhabdus* sp. strain M41 (GenBank accession number NZ_CP014545).

All whole-genome-sequenced species in the order *Sphingomonadales* available on the NCBI’s RefSeq ftp site (ftp://ftp.ncbi.nlm.nih.gov/genomes/refseq/bacteria/; accessed 4 July 2018) were then included with strain SMR4y in a phylotaxonomic analysis using PhyloPhlAn version 0.99 (10). This showed strain SMR4y to be a sister to *Sphingorhabdus* sp. strain YGSMI21; these two strains, along with *Sphingorhabdus* sp. strain M41, form a clade with 100% bootstrap support. Considering the results of the above-described analyses, we placed strain SMR4y in the genus *Sphingorhabdus*.

### Data availability.

This whole-genome project has been deposited in GenBank under the accession number CP022336 as part of BioProject number PRJNA380207. The raw reads have been deposited in the NCBI Sequence Read Archive under the accession number SRR5811295.
